# Prevalence and associated factors of social frailty among older adults in nursing homes: a cross-sectional study

**DOI:** 10.3389/fpubh.2025.1742570

**Published:** 2025-12-18

**Authors:** Ting-Ting Wang, Yang Zhao, Ling-Na Kong, Jun Yang, Wen-Xin Wang, Lu Chen, Ju Qiu, Fen Xie, Ying Huang, Li-Zhen Wang

**Affiliations:** 1 School of Nursing, Chongqing Medical University, Chongqing, China; 2 Department of General Practice, The First Affiliated Hospital of Chongqing Medical University, Chongqing, China; 3 Department of Breast and Thyroid Surgery, The First Affiliated Hospital of Chongqing Medical University, Chongqing, China

**Keywords:** associated factors, nursing homes, older adults, prevalence, social frailty

## Abstract

**Aims:**

To investigate the prevalence of social frailty among older adults in nursing homes, and to explore the associated factors of social frailty.

**Design:**

A cross-sectional study.

**Methods:**

From September to December 2024, a convenience sample of 447 older adults was recruited from nine nursing homes in Chongqing, China. Social frailty was assessed using the HALFT scale, with a score of ≥ 3 indicating the presence of social frailty, Data were collected on demographic and disease characteristics, loneliness, depression, and social network. Descriptive statistics, univariate analysis and multivariate logistic regression analysis were applied for data analysis.

**Results:**

The prevalence of social frailty was 23.9% among older adults in nursing homes. Multivariate logistic regression analysis indicated that education level (OR = 0.306, 95% CI:0.099–0.945, *p* < 0.001), willingness to reside (OR = 2.816, 95% CI:1.147–6.915, *p* = 0.024), loneliness (OR = 1.354, 95% CI:1.214–1.510, *p* < 0.001), depression (OR = 1.753, 95% CI:1.229–2.499, *p* = 0.002), and social network (OR = 0.955, 95% CI:0.914–0.999, *p* = 0.043) were independent factors associated with social frailty.

**Conclusion:**

Social frailty is a common problem among older adults in nursing homes. Healthcare providers should develop preventive strategies based on these factors to reduce the onset and progression of social frailty among older adults in nursing homes.

## 1 Background

Social frailty is defined as the lack or loss of social resources, behaviors, activities, and self-management skills (1). It is associated with adverse outcomes such as depression, cognitive impairment, and disability, reducing the quality of life in older adults and imposing a heavy burden on families and healthcare systems (2). The confined living environment, stringent management protocols, and limited activity arrangements in nursing homes may diminish opportunities for social participation and autonomy among older adults (3). This can lead to negative attitudes toward social participation, reluctance to engage in communication, and unmet social needs, increasing the risk of social frailty (4). Research demonstrated that the high risk of social frailty among older adults in nursing homes is positively associated with adverse outcomes (5). This risk translates into a substantial societal burden through pathways such as individual health deterioration, increased family caregiving pressure, and elevated healthcare system costs (5). Thus, it is essential to implement early screening for social frailty and identify its associated factors specifically among older adults in nursing homes to develop targeted interventions.

Social frailty is influenced by various factors, including psychosocial and demographic elements (1). Loneliness is associated with social frailty and is characterized by subjective distress, often manifesting as alienation in close relationships and a shrinking social network (6). Studies have shown that higher levels of loneliness are significantly linked to an increased likelihood of social frailty in older adults (7). Lonely individuals are more likely to adopt unhealthy lifestyles, reduce social participation, and thus enter a vicious cycle of social frailty (8). Depression is characterized by persistent low mood, loss of interest, dysfunction in social roles, and impaired cognitive adaptability (2). Research indicates that depression exacerbates the development of social frailty and is closely related to components such as living alone and reduced social support (9, 10). Furthermore, social network is a circle of people who are connected to each other through various relationships (11). Although a direct link between social network and social frailty has not been established, some studies suggest that lack of social network may accelerate the decline in physical and cognitive function, thereby impairing the ability to maintain social connections and facilitating the progression of social frailty (12). Demographic factors such as gender, age, and education have also been associated with social frailty, although findings remain inconsistent (13).

It is worth noting that although existing studies have identified associated factors for social frailty, most of the evidence pertains to older adults living in communities. Comparatively little attention has been paid to those older adults in nursing homes. Therefore, the purpose of this study is to enrich the research on social frailty by analyzing the prevalence and associated factors of social frailty among older adults in nursing homes. The results of this study can provide reference to improve the quality of life of older adults with social frailty.

## 2 Methods

### 2.1 Study design and participants

A cross-sectional and descriptive design was adopted. This study employed a convenience sampling method to recruit older adults from nine nursing homes in Chongqing, China. The inclusion criteria were as follows: (1) age of years ≥ 60, (2) length of stay ≥ 3 months, (3) clear consciousness and regular language expression, and (4) informed consent. We excluded participants if they with dementia, communication difficulties, and other severe health conditions that may affect them to complete questionnaires.

The required sample size was calculated using the formula for sample size in an observational study: N = *Z*^2^_1-*α*/2_ × *P*(1-*P*)/*δ*^2^ (14), with a significance level of *α* = 0.05 (95% confidence). When the allowable error (δ) was set at 0.05 and the prevalence of social frailty (*P*) of 36.6% (15), the sample size was calculated as: N = (1.96)^2^ × 0.366 × (1–0.366)/(0.05)^2^ ≈ 357. To account for a 20% potential non-response or sampling bias (16), the final target sample was 447 (357÷0.8 ≈ 447).

### 2.2 Instruments

The questionnaire consisted of five sections: demographic and disease characteristics list, the HALFT Scale, the 6-item UCLA Loneliness Scale, the Geriatric Depression Scale-5, and the National Center for Geriatrics and Gerontology Social Network Scale. The demographic characteristics included age (years), sex (male or female), marital status (married or unmarried), education level (illiterate, primary school, junior middle school, senior high school, college or above), living arrangement (living alone, with spouse, or with others), willingness to reside (voluntary or involuntary), and length of residence (<1, 1–3, or >3 years). Disease characteristics included the number of chronic diseases (0, 1–2, 3–4, and ≥5 types), visual impairment (yes or no), and hearing impairment (yes or no).

Social frailty was assessed using the HALFT scale (17), which was originally developed and validated in a Chinese cultural context. The scale covers five items: inability to help others, limited social participation, loneliness, financial difficulty, and not having anyone to talk to. Ranges from 0 to 5 for social frailty, with a score ≥ 3 indicating its presence. To confirm its suitability for our specific study population (older adults in Chongqing nursing homes), we conducted a preliminary test (pilot study) prior to the main investigation. The internal consistency in this pilot test (n = 30) was acceptable, with a Cronbach’s *α* of 0.644, which is consistent with the reported value of 0.635 in the original Chinese validation study (7). In the present main study, the Cronbach’s α was 0.629.

It is important to note that while the HALFT scale contains a single-item indicator of loneliness, its primary function in this study is to generate a composite score for the outcome of social frailty. In contrast, loneliness as a psychosocial factor was measured independently using the validated 6-item UCLA Loneliness Scale (ULS-6). This approach allows for a more precise and multidimensional assessment of loneliness as an exposure variable in our analysis, distinct from its role as one component within the social frailty outcome.

Loneliness was measured by the 6-item UCLA Loneliness Scale (ULS-6) (18). It consists of six items, and each item is rated on a 4-point Likert scale ranging from 1 (never) to 4 (always). The total score ranges from 6 to 24, with higher scores indicating greater perceived loneliness. The Cronbach’s *α* of ULS-6 was 0.810 among older Chinese (18). In this study, the Cronbach’s α was 0.946.

Depression was evaluated using the Geriatric Depression Scale-5 (GDS-5) (10). It includes five items, each scored as 0 or 1, with a total score ranging from 0 to 5. Scores of 0–1 are considered normal, and ≥2 suggest depression, with higher total scores indicating more severe depression. The Cronbach’s *α* for GDS-5 was 0.800 in the original study (19), and it was 0.704 in this study.

Social network was measured by the National Center for Geriatrics and Gerontology Social Network Scale (NCGG-SNS) (20). It consists of four domains: face-to-face/non-face-to-face interactions with family/friends. Each domain score is calculated by multiplying the frequency and satisfaction ratings for each item. The total score ranges from 0 to 64, with higher scores indicating a better social network. The original test–retest reliability and Cronbach’s *α* were 0.960 and 0.690, respectively (20). In this study, the Cronbach’s α was 0.803.

### 2.3 Data collection

Data were collected from September to December 2024 using a self-report structured questionnaire. Trained researchers recruited older adults in nursing homes who met the inclusion criteria, and explained the purpose and content of this study. Eligible participants who agreed to participate were asked to complete a paper-and-pencil questionnaire. Two trained researchers initially cross-checked each completed questionnaire for obvious errors (e.g., missing items, logical inconsistencies). If a potential error was identified, a third researcher, who was blinded to the initial assessment and the specific concerns of the first two researchers, conducted a follow-up. This third researcher neutrally re-asked only the specific item(s) in question, without any leading information, and recorded the new response. Only this independently verified response was entered into the final database.

### 2.4 Data analysis

IBM SPSS 25.0 was used for statistical analysis. The participants’ social frailty status was classified as social frailty or non-social frailty. Categorical data were presented as frequency (percentage), while continuous data were presented as mean ± standard deviation. The selection of candidate predictors for the multivariable model followed a two-step process. First, potential factors were identified *a priori* based on a review of relevant literature and clinical relevance, as reflected in the construction of the general characteristics’ questionnaire. Second, to refine the model and avoid overfitting, these candidate variables were subjected to univariate analysis. Specifically, the chi-square test (for categorical variables) or the t test (for continuous variables) was used to examine their initial association with social frailty. All variables showing an association with social frailty at *p* < 0.05 in the univariate analyses were subsequently entered into a multivariable binary logistic regression model to identify factors independently associated with social frailty, while adjusting for the effects of other variables. Results are presented as odds ratios (OR) with 95% confidence intervals (CI). The Hosmer-Lemeshow test was performed to assess the goodness of fit of the logistic regression model (21).

### 2.5 Ethical statement

This study was approved by the Ethics Committee of the First Affiliated Hospital of Chongqing Medical University (2024–149-01). The study was conducted in accordance with the principles of the Declaration of Helsinki. Written informed consent was obtained from all participants after a comprehensive explanation of the study, ensuring autonomous decision making and voluntary engagement in the research.

## 3 Results

### 3.1 Demographic characteristics and social frailty status of participants

A total of 470 questionnaires were distributed during the study. 23 participants were excluded due to refusal to complete (*n* = 14) or missing data on key variables (*n* = 9), resulting in a final valid sample of 447 participants (valid response rate: 95.1%). Among the 447 participants, 107 (23.9%) met the criteria for social frailty (HALFT scale ≥3). The mean age was 84 ± 6 years, and 277 (62%) were female. Over 60% of participants had an education level of junior middle school or below. The majority (78.5%) did not have a partner. A total of 47.2% lived with others, and more than half (52.3%) had been admitted to the nursing home involuntarily. A total of 135 participants had lived in the nursing home for more than 3 years. Visual impairment was reported in 169 individuals (37.8%), and hearing impairment in 98 (21.9%). The mean loneliness score was 9.70 ± 4.19, the mean depression score was 0.56 ± 1.05, and the mean social network score was 18.23 ± 10.89 (see Table 1).

**Table 1:** General demographic data and univariate analyses (*N* = 447).

Variables	Total (*n* = 447)	Non-social frailty (*n* = 340)	Social frailty (*n* = 107)	*χ*^2^/t	*p*
Age, years, $\bar{x}$ ±*s*	84.38 ± 6.44	84.85 ± 6.09	82.91 ± 7.27	−2.668^b^	0.008*
Sex, *n* (%)				15.615^a^	<0.001*
Male	170 (38.0)	112 (65.9)	58 (34.1)		
Female	277 (62.0)	228 (82.3)	49 (17.7)		
Marital status, *n* (%)				3.550^a^	0.060
Married	96 (21.5)	80 (83.3)	16 (16.7)		
Unmarried	351 (78.5)	260 (74.1)	91 (25.9)		
Education level, *n* (%)				74.406^a^	<0.001*
Illiterate	100 (22.4)	49 (49.0)	51 (51.0)		
Primary school	116 (26.0)	80 (69.0)	36 (31.0)		
Junior middle school	86 (18.6)	74 (89.2)	9 (10.8)		
Senior high school	102 (22.8)	92 (90.2)	10 (9.8)		
College or above	46 (10.3)	45 (97.8)	1 (2.2)		
Living arrangement, *n* (%)				6.712^a^	0.035*
Living alone	152 (34.0)	117 (77.0)	35 (23.0)		
Living with spouse	84 (18.8)	72 (85.7)	12 (14.3)		
Living with others	211 (47.2)	151 (71.6)	60 (28.4)		
Willingness to reside, *n* (%)				63.789^a^	<0.001*
Voluntary	213 (47.7)	198 (93.0)	15 (7.0)		
Involuntary	234 (52.3)	142 (60.7)	92 (39.3)		
Length of residence, *n* (%)				0.363^a^	0.834
<1 year	129 (28.9)	98 (76.0)	31 (24.0)		
1–3 years	183 (40.9)	137 (74.9)	46 (25.1)		
>3 years	135 (30.2)	105 (77.8)	30 (22.2)		
Number of chronic diseases, *n* (%)				0.210^a^	0.976
0	70 (15.7)	54 (77.1)	16 (22.9)		
1–2	205 (45.9)	154 (75.1)	51 (24.9)		
3–4	139 (31.1)	107 (77.0)	32 (23.0)		
≥5	33 (7.4)	25 (75.8)	8 (24.2)		
Visual impairment, *n* (%)				22.059^a^	<0.001*
Yes	169 (37.8)	108 (63.9)	61 (36.1)		
No	278 (62.2)	232 (83.5)	46 (16.5)		
Hearing impairment, *n* (%)				9.561^a^	0.002*
Yes	98 (21.9)	63 (64.3)	35 (35.7)		
No	349 (78.1)	277 (79.4)	72 (20.6)		
Loneliness (score, $\bar{x}$ ± *s*)	9.70 ± 4.19	8.17 ± 2.81	14.53 ± 4.19	−12.612^b^	<0.001*
Depression (score, $\bar{x}$ ± *s*)	0.56 ± 1.05	0.24 ± 0.65	1.55 ± 1.41	−10.957^b^	<0.001*
Social network (score, $\bar{x}$ ± *s*)	18.23 ± 10.89	20.80 ± 10.38	10.06 ± 8.13	−0.929^b^	<0.001*

a Chi-square test used for comparing categorical variables between groups.

b *t*-test, used for comparing continuous variables between groups.

$\bar{x}$ , mean; s, standard deviation. *Significant differences between groups.

### 3.2 Univariate analysis of social frailty among older adults in nursing homes

Univariate analysis identified statistically significant differences in age, sex, educational level, living arrangement, willingness to reside, visual impairment, hearing impairment, loneliness, depression, and social network among older adults with varying levels of social frailty in nursing homes (*p* < 0.05). No significant differences were observed in marital status, length of resident, or number of chronic diseases (*p* > 0.05), as detailed in Table 1.

### 3.3 Multivariate logistic regression analysis of social frailty among older adults in nursing homes

The measurement data of the independent variables are entered with the original value, and the other values are shown in Table 2. The results showed that education level, willingness to reside, loneliness, depression, and social network were independently associated with social frailty among older adults in nursing homes. The Hosmer and Lemeshow test showed *χ*^2^ = 1.739 and *p* = 0.988, indicating a good fit of the model. The model can correctly predict 89.3% of the study participants with high accuracy. See Table 3 for details.

**Table 2:** Assignment table of argument variables.

Variables	Assignment
Age	Original value entry
Marital status	Unmarried = 0; Married = 1
Education level	Illiterate = 0; Primary school = 1; Junior middle school = 2; Senior high school = 3; College or above = 4
Living arrangement	Living alone = 0; Living with spouse = 1; Living with others = 2
Willingness to reside	Active = 0; Passive = 1
Visual impairment	No = 0; Yes = 1
Hearing impairment	No = 0; Yes = 1
Loneliness	Original value entry
Depression	Original value entry
Social network	Original value entry

**Table 3:** Multivariate logistic regression analysis of social frailty (N = 447).

Variables	*B*	*SE*	*Waldχ^2^*	*p*	*OR*	*95% CI*
Age	0.010	0.028	0.120	0.729	1.010	0.956–1.066
Sex	−0.130	0.374	0.138	0.710	0.870	0.419–1.810
Education level						
Illiterate	–	–	13.139	0.011*	–	–
Primary school	−0.416	0.416	1.229	0.268	0.630	0.279–1.425
Junior middle school	−1.805	0.565	10.193	0.001*	0.164	0.054-0.498
Senior high school	−1.183	0.575	4.238	0.040*	0.306	0.099-0.945
College or above	−2.462	1.259	3.827	0.050*	0.085	0.007-1.005
Living arrangement						
Living alone	–	–	1.506	0.471	–	–
Living with spouse	−0.510	0.569	0.802	0.371	0.601	0.197–1.833
Living with others	−0.467	0.406	1.329	0.249	0.627	0.283–1.387
Willingness to reside	1.035	0.458	5.103	0.024*	2.816	1.147-6.915
Visual impairment	0.691	0.373	3.423	0.064	1.996	0.960–4.150
Hearing impairment	−0.038	0.423	0.008	0.928	0.962	0.420–2.205
Loneliness	0.303	0.056	29.601	<0.001*	1.354	1.214-1.510
Depression	0.561	0.181	9.615	0.002*	1.753	1.229-2.499
Social network	−0.046	0.023	4.088	0.043*	0.955	0.914-0.999

B, partial regression weight; SE, standard error; Waldχ^2^, Wald chi-square statistic; OR, Odds ratio; CI, confidence interval.

*Significant differences between groups.

## 4 Discussion

This study aimed to investigate the prevalence and associated factors of social frailty among older adults in nursing homes. The results showed that social frailty was common in this population. Multivariate logistic regression analyses indicated that education level, willingness to reside, loneliness, depression, and social network were independently associated with social frailty among older adults in nursing homes. These findings may help to identify populations at high risk for social frailty and provide a basis for future research. It is noteworthy that in our sample, the univariate analysis suggested a lower average age among the socially frail. This may be relatively young older adults may have experienced acute health crises, leading to sudden disruptions in their established social roles and external social networks. They face more pronounced psychological dissonance, lack proactive social support, and become trapped in a state of social isolation.

The results of this study indicate that the prevalence of social frailty was 23.9% among older adults in nursing homes, higher than the 7.7% reported among community-dwelling older adults (17). This discrepancy may be due to differences in daily lifestyles and living environments between nursing homes and community-dwelling older adults. Older adults in nursing homes experience prolonged separation from family and friends, live in closed and monotonous environments, and have limited daily activities and social interactions (22). These conditions may contribute to negative emotional states such as loneliness and depression. Previous studies have identified an association between negative emotions and social frailty in older adults (23). Therefore, healthcare providers should pay greater attention to social frailty among older adults in nursing homes, identify and screen high-risk groups for social frailty as early as possible, and implement relevant intervention measures to prevent adverse outcomes.

Education level was associated with social frailty among older adults in nursing homes, consistent with previous study (24). Education level has been recognized as a key factor in promoting social participation among older adults. Studies have shown that older adults with higher levels of education are associated with more positive attitudes toward life (25). Moreover, higher education levels are associated with greater learning capability, a stronger inclination to build diverse interest networks, and a greater tendency to use diverse social strategies (e.g., digital technologies) to maintain social connections. These attributes may collectively contribute to a dynamic defense system against social frailty. In addition, Irshad et al. (26) found higher education levels to be a protective factor for social frailty, which is consistent with the present study. Older adults with higher education levels possess higher health information literacy and are able to recognize, interpret, and apply knowledge about disease prevention and health management. They also actively participate in social activities, which is associated with better social functioning and a lower likelihood of social frailty. Therefore, healthcare providers should focus on older adults with lower education levels and implement individualized interventions, such as health education, to prevent or reduce the incidence of social frailty.

This study demonstrated that the willingness to reside in a nursing home was an important associated factor of social frailty among older adults, and that the risk of social frailty is 2.8 times higher among older adults who enter nursing homes involuntarily than those who choose to do so voluntarily. This may be because involuntary older adults often experience a strong sense of psychological deprivation and loss of control, which may weaken their motivation and ability to establish new social relationships (27). In addition, involuntary residents often lack adequate psychological preparation before admission. Following the disruption of their existing social networks, they may find it difficult to rebuild effective support systems within institutional settings, and report feelings of isolation and abandonment (28). Moreover, involuntary placement often reflects a deficient or absent family support system. These older adults are more likely to become trapped in a vicious cycle… which further reduces their social participation and is associated with a higher likelihood of social frailty (22). It is recommended that health care providers assess older adults’ willingness to enter nursing homes and implement social adjustment interventions for involuntary residents. These may include establishing structured health management systems and organizing socially engaging activities to help older adults adapt to institutional life, foster a sense of belonging and control, and potentially mitigate social frailty.

Loneliness is an independent associated factor for social frailty, which was supported by the previous finding (7). Related studies have shown that loneliness is a common problem among older adults in nursing homes (6). Older adults in nursing homes are more vulnerable to negative psychological responses such as loneliness. This may be due to separation from family members and the challenge of adapting to new living environments and social networks, which can limit their social participation (29). In our sample, more than half of the older adults were unmarried or widowed, and 34.8% lived alone. Lonely older adults often show reluctance to communicate with others, which objectively leads to social isolation and reduced participation. Subjectively, they may feel excluded by others, which is concurrently linked to more severe social frailty (8). It is recommended that health care providers increase early screening for older adults at risk of loneliness and actively establish teams of mental health professionals to provide personalized psychological counselling and support services.

Depression is associated with an increased risk of social frailty among older adults in nursing homes, which is consistent with previous findings in community-dwelling older adults (9, 30). Depression is a common psychological problem among older adults in nursing homes. This population may be vulnerable to depressive symptoms due to discomfort with the new environment, lack of family support, and financial pressures (3, 22). Studies have shown that common symptoms of depression, such as insomnia, apathy, and decreased motivation, can reduce both the willingness and ability of older adults to participate in activities (10). Older adults with depression have lower life satisfaction, which is correlated with fewer social and recreational activities, reduced participation, and greater susceptibility to social frailty (9, 31). Therefore, healthcare providers should monitor the mental health of older adults, identify depressive symptoms at an early stage, and implement targeted interventions. In addition, facilitating emotional support activities that encourage interpersonal interaction may help alleviate negative emotions and potentially mitigate social frailty.

This study found that older adults in nursing homes with higher social network scores had a lower risk of social frailty. Previous studies have shown that older adults in nursing homes have a higher risk of reduced social network (32). Narrower social circles and limited social interactions objectively characterize reduced social network (11). Decreased social network is associated with physical frailty, cognitive decline, and negative emotions, which can adversely affect the health of older persons (33). These effects can limit their ability to engage in physical activities, reduce opportunities for social participation, which is linked to a higher risk of social frailty (34). It is suggested that health care providers pay attention to the mental well-being of older adults. In addition to improving infrastructure and medical services, they should strengthen psychological care measures, regularly conduct communication activities, encourage the active participation of older adults and their family members, maintain or expand their social networks, promote social interaction and participation, and enhance their sense of social support, which may help mitigate social frailty.

## 5 Limitations

This study has several limitations. First, the cross-sectional nature of this study precludes the establishment of causal relationships. Second, this study primarily focuses on the impact of sociodemographic and psychological factors on social frailty; thus, other relevant factors such as cognitive status, physical function, and sleep quality were not included. Third, self-report survey methods are susceptible to subject recall bias and social desirability bias, which may affect the accuracy of reported information. Therefore, future study may conduct a series of multicenter, large-sample longitudinal studies to verify and expand the relevant results. Alternatively, risk prediction models could be developed to precisely calculate an individual’s risk of developing social frailty.

## 6 Conclusion

The prevalence of social frailty among older adults in nursing homes was 23.9%. Several factors, such as education level, willingness to reside, loneliness, depression, and social network, significantly influence the severity of social frailty. These findings underscore the importance of routine screening for social frailty among older adults in nursing homes, particularly those at higher risk, such as individuals with limited literacy, those who were involuntarily admitted, or those with poor psychological resilience. Therefore, healthcare professionals should perform a comprehensive assessment of each older adult’s specific circumstances. Based on these assessments, they can then implement evidence-based, targeted interventions designed to reduce or delay the development and progression of social frailty.

## Data Availability

The original contributions presented in the study are included in the article/supplementary material, further inquiries can be directed to the corresponding author.
